# Social Media Platforms and Sustainable Tourism Choices: The Role of Online Review Credibility and Cognitive Processing

**DOI:** 10.3390/bs16060919

**Published:** 2026-06-03

**Authors:** Anar Eminov, Natavan Namazova, Zivar Zeynalova, Elkhan Richard Sadik-Zada

**Affiliations:** 1 Department of Management, Mingachevir State University, Mingachevir AZ4500, Azerbaijan; anar.eminov@mdu.edu.az; 2 Department of Economics, Mingachevir State University, Mingachevir AZ4500, Azerbaijan; natavan.namazova@mdu.edu.az; 3 Department of Digital Economy and Financial Technologies, Azerbaijan Technical University, Baku AZ1073, Azerbaijan; 4 Institute of Development Research and Development Policy, Ruhr-University of Bochum, 44801 Bochum, Germany; 5 University College, Korea University, 145 Anam-ro, Seongbuk District, Seoul 02841, Republic of Korea; 6 Center for Studies on European Economy, Azerbaijan State University of Economics (UNEC), Istiqlaliyyat Ave. 6, Baku AZ1001, Azerbaijan

**Keywords:** user-generated content, perceived credibility, social media, ecological awareness, cognitive processing bias, sustainable tourism, moderated mediation

## Abstract

This study examines how social media–driven information environments shape pro-environmental travel decision-making within the broader context of sustainable tourism and digital information expansion. It aims to explain how informational, cognitive, and evaluative mechanisms jointly influence behavioral intention by integrating perceived trustworthiness of user-generated content, ecological awareness, attitude formation, and cognitive processing bias within a moderated mediation framework. Data were collected from 315 respondents in Azerbaijan through a structured survey and analyzed using structural equation modeling to test both mediating and conditional relationships. The results show that perceived trustworthiness of user-generated content significantly strengthens attitude formation, which is the strongest predictor of behavioral intention. Trust also has a direct effect, but its influence mainly operates through evaluative pathways. Ecological awareness partially mediates the relationship between trust and intention, indicating multiple parallel mechanisms. Furthermore, cognitive processing bias weakens the effect of trust on attitude and reduces indirect effects. Overall, the findings suggest that sustainable tourism behavior is driven more by credibility-based cognitive evaluation than by mere information availability, highlighting the importance of credibility-enhancing and cognitively effective communication strategies.

## 1. Introduction

The rapid expansion of digital technologies and the widespread use of social media platforms have fundamentally transformed tourism. They have changed how tourists’ access, evaluate, and use information in the decision-making process. Social media platforms have become dominant channels for information search and travel planning, significantly influencing tourists’ perceptions and behaviors (Xiang & Gretzel, 2010; Hussain et al., 2024; Kelmendi, 2025). In particular, user-generated content and electronic word-of-mouth (e-WOM) play a crucial role in shaping tourism decisions. They provide experiential and peer-based information (Cheung et al., 2008; Erkan & Evans, 2016; Saif & Nofal, 2026).

In such digitally mediated environments, however, the process of information evaluation is not purely rational. Tourists are frequently exposed to large volumes of content, often presented through algorithm-driven feeds and popularity cues. As a result, they tend to rely on heuristic signals, selective attention, and simplified processing strategies when interpreting online information. These tendencies, conceptualized as cognitive processing bias, influence not only how information is interpreted but also whether it is translated into meaningful evaluations and behavioral intentions. Under conditions of information overload, such biases may systematically distort or weaken the impact of otherwise credible information.

This cognitive dimension becomes particularly important when considering the role of online reviews. Within the same digital environment, online reviews have emerged as one of the most influential sources of tourism-related information. Tourists rely on these reviews to evaluate service quality, trustworthiness, and destination attractiveness, with their effectiveness largely depending on perceived credibility (Filieri & McLeay, 2014; Sparks & Browning, 2011; Ayeh et al., 2013; Filieri et al., 2018). However, the influence of such information cannot be understood independently of how it is cognitively processed. In other words, credibility does not operate in isolation; its impact depends on the way individuals interpret and evaluate the information they encounter.

This issue becomes more important in the context of sustainable tourism. In this study, sustainable tourism choices refer to tourists’ preferences and intentions toward environmentally responsible tourism services, destinations, and consumption practices that support long-term environmental sustainability and reduce negative environmental impacts. Unlike general pro-environmental attitudes, sustainable tourism choices are viewed as behavioral outcomes shaped by credibility perceptions, cognitive evaluation, environmental awareness, and digital information-processing mechanisms. Thus, sustainable tourism is considered not as a separate tourism category, but as a value-oriented behavioral approach that influences decision-making across different tourism contexts.

Environmental claims are often complex and difficult for tourists to verify directly, which increases the importance of trust and cognitive evaluation (Chao & Zhang, 2025; Han, 2015). At the same time, sustainable tourism has become a major priority due to growing environmental problems and the need for responsible consumption. Although environmental awareness has increased, a gap still exists between positive environmental attitudes and actual behavioral intentions. This problem is especially visible in tourism, where decisions are complicated, information is fragmented, and sustainability claims are often unclear (Nekmahmud et al., 2022; J. Li et al., 2024; Rahman & Mia, 2025; García-García et al., 2023). Therefore, the main challenge is not only access to information, but also how individuals process and interpret sustainability-related information in digital environments. In response to this issue, the present study integrates perceived credibility of online reviews, environmental awareness, and cognitive processing bias within a moderated mediation framework to explain sustainable tourism decision-making.

From a theoretical perspective, the Theory of Planned Behavior (TPB) explains how attitudes shape behavioral intentions in environmentally responsible contexts (Han et al., 2010; C. Wang et al., 2018; Wut et al., 2023).

Although the Theory of Planned Behavior traditionally includes attitude, subjective norms, and perceived behavioral control as predictors of behavioral intention, the present study does not aim to test the full TPB structure. Instead, it adopts an attitude-centered adaptation of TPB specifically suited to digitally mediated tourism environments. The study focuses on attitude because the primary objective is to examine how credibility-based digital information and cognitive processing mechanisms shape evaluative judgments and behavioral intention. In contrast, subjective norms and perceived behavioral control are not incorporated, as the emphasis of the model is placed on informational credibility and individual cognitive evaluation rather than social pressure or resource-related behavioral constraints. This selective integration is consistent with prior studies that employ partial TPB frameworks to investigate context-specific cognitive and informational mechanisms in digital decision-making environments.

At the same time, TPB largely assumes stable and rational cognitive processing, which may not fully capture the dynamics of decision-making in digital environments. In parallel, the e-WOM literature emphasizes the role of online information and perceived credibility, yet it often treats information processing as relatively uniform and linear (Rahman & Mia, 2025; Nguyen, 2025; Rao et al., 2025; Boneta-Ruiz et al., 2025; Hu et al., 2025; Wei et al., 2025; Kelmendi, 2025; Hussain et al., 2024; Xu et al., 2021; Ogiemwonyi et al., 2023). When considered together, these perspectives reveal an important theoretical limitation: while TPB explains attitudinal mechanisms and e-WOM explains informational influence, neither framework sufficiently accounts for variability in cognitive processing under digital conditions.

More specifically, prior TPB and e-WOM studies generally assume that individuals process digital information in relatively stable and rational ways. However, in digitally saturated environments characterized by information overload, algorithmic exposure, and heuristic evaluation, the influence of credible information may vary substantially across individuals depending on their cognitive processing tendencies. In this context, Cognitive Processing Bias is conceptualized not merely as an additional predictor, but as a boundary condition that determines whether and to what extent credibility-based information is translated into sustainable tourism attitudes and behavioral intentions.

To address this limitation, the present study integrates TPB and e-WOM perspectives with a cognitive processing lens by explicitly incorporating Cognitive Processing Bias as a conditional factor. The study conceptualizes sustainable tourism decision-making as a cognitively mediated process in which social media platforms shape perceived credibility, which subsequently influences attitudes and behavioral intentions. Environmental awareness functions as a mediating cognitive mechanism facilitating the internalization of sustainability-related information, while Cognitive Processing Bias influences the extent to which credible information is systematically evaluated and translated into attitudinal outcomes.

Despite this integrative perspective, an important gap remains. First, limited attention has been paid to the interaction between digital information environments and cognitive processing mechanisms in shaping sustainable tourism choices. Second, the indirect effects of perceived credibility through psychological constructs such as attitude and environmental awareness have not been sufficiently integrated within a unified analytical framework. Third, the role of cognitive processing bias as a boundary condition in digital decision-making remains largely overlooked. Addressing these gaps is essential for explaining why credible information does not always lead to consistent behavioral outcomes.

Accordingly, the objective of this study is to examine the mechanisms through which social media platforms influence tourists’ behavioral intention toward sustainable tourism choices, with particular emphasis on perceived credibility, attitude, environmental awareness, and cognitive processing bias. The study addresses the following research questions:

RQ1: How do social media platforms influence perceived credibility of online reviews and, subsequently, tourists’ attitudes toward sustainable tourism?
RQ2: How do perceived credibility, attitudes, and environmental awareness jointly shape behavioral intention toward sustainable tourism choices, including the mediating role of environmental awareness?
RQ3: How does cognitive processing bias conditionally influence the relationship between perceived credibility, attitude, and behavioral intention in the context of sustainable tourism?

This study contributes to the literature by introducing a cognitively conditioned extension of TPB and e-WOM models, demonstrating that the impact of information credibility on behavior depends on individual-level cognitive processing conditions rather than operating uniformly across users. By integrating informational (credibility), cognitive (environmental awareness), and evaluative (attitude) mechanisms within a moderated mediation framework, the study provides a more precise explanation of variability in sustainable tourism behavior.

From a practical perspective, the findings offer actionable insights for multiple stakeholder groups. For tourism service providers, enhancing the credibility of user-generated content through verified reviews and transparent sustainability claims can strengthen consumer trust and influence decision-making. For digital platform designers, the results highlight the importance of algorithmic curation mechanisms that prioritize credible and sustainability-oriented content over purely engagement-driven signals. For destination managers and policymakers, investing in environmental awareness campaigns can improve individuals’ ability to process sustainability-related information more systematically, thereby increasing the effectiveness of sustainability initiatives.

## 2. Literature Review and Hypotheses

### 2.1. Social Media Platforms and Perceived Credibility of Online Reviews

The proliferation of social media platforms has fundamentally reshaped how tourists search for, interpret, and evaluate travel-related information. Unlike traditional communication channels, social media environments operate as interactive and socially embedded systems. In these systems, user-generated content is continuously created, evaluated, and redistributed (Xiang & Gretzel, 2010; Cheung et al., 2008; X. Leung et al., 2015). This transformation is particularly relevant in tourism, where experiential information and peer-generated evaluations significantly influence decision-making processes.

However, the influence of social media on decision-making cannot be understood solely in terms of information availability. Rather, it depends on how information is evaluated within digitally mediated environments. From a theoretical perspective, credibility is not an inherent property of information but a socially constructed perception shaped by contextual and interactional cues (Filieri et al., 2018; Lou & Yuan, 2019; Almohammed et al., 2025). Social media platforms provide such cues through mechanisms like ratings, likes, comments, and peer feedback. These act as heuristic signals that guide credibility assessments.

Importantly, these mechanisms operate through both systematic and heuristic processing pathways. According to dual-process theories, individuals evaluate information in two ways. They either use effortful, analytical processing or rely on simplified heuristic cues (Chaiken, 1980; Petty & Cacioppo, 1986). In social media environments, information volume is high and attention is limited. Therefore, heuristic cues such as popularity, source familiarity, and social endorsement often become dominant (Dwivedi et al., 2021; Rosillo-Díaz et al., 2024; Harrigan et al., 2017). As a result, credibility perceptions are shaped not only by message quality but also by platform-specific signals.

This duality explains why social media environments can simultaneously enhance and distort credibility perceptions. On one hand, interactive features increase transparency and enable collective validation, strengthening perceived reliability. On the other hand, reliance on heuristic cues may lead to superficial evaluations. Highly visible or popular content may be perceived as more credible, regardless of its actual quality.

In sustainable tourism contexts, this issue becomes even more pronounced. Sustainability-related claims are often complex, abstract, and difficult to verify directly, increasing reliance on externally provided cues (Boneta-Ruiz et al., 2025; Hidalgo-Alcazar et al., 2021; Lee, 2011). Consequently, social media platforms play a critical role not only as information sources. They also act as credibility-shaping environments where perceptions of authenticity and trustworthiness are formed.

Based on this perspective, the study conceptualizes social media platforms as evaluative environments. These environments shape how credibility is formed through the interaction of content and social signals. Accordingly, the following hypothesis is proposed:

**H1.**  *The use of social media platforms has a significant positive effect on the perceived credibility of online reviews related to sustainable tourism services.*

### 2.2. Perceived Credibility of Online Reviews and Attitude Toward Sustainable Tourism

Perceived credibility plays a central role in determining how individuals process, internalize, and evaluate information. Within the framework of information adoption theory, credible information is more likely to be perceived as diagnostic and relevant. As a result, it influences belief formation and evaluative judgments (Filieri, 2015; Cheung et al., 2008). However, this relationship is not purely informational; it involves deeper cognitive and evaluative mechanisms.

From a theoretical standpoint, credibility functions as both an uncertainty-reduction mechanism and a legitimization process. In tourism contexts, where service quality and destination attributes are often uncertain, credible online reviews reduce perceived risk and enhance confidence in decision-making (Ayeh et al., 2013; Elizabeth et al., 2025). In sustainable tourism contexts, this role becomes more critical because environmental claims are often intangible and difficult to verify directly.

Beyond reducing uncertainty, credibility also serves to legitimize sustainability-related information. When tourists perceive online reviews as credible, they are more likely to accept sustainability claims as meaningful and trustworthy. They also integrate these claims into their evaluative framework. This process can be explained through cognitive consistency theory, where individuals align their attitudes with information perceived as reliable and relevant.

Importantly, the credibility–attitude relationship is not always linear or stable. Prior studies suggest that this relationship depends on how information is processed and interpreted (Hidalgo-Alcazar et al., 2021). For example, under conditions of high cognitive effort, individuals are more likely to engage in systematic evaluation. This leads to stronger and more stable attitudes. In contrast, under heuristic processing, credibility may influence attitudes more superficially.

Thus, perceived credibility should be understood not merely as an informational input but as a cognitive trigger that activates evaluative processes. In sustainable tourism, this mechanism is particularly important because attitudes toward sustainability are shaped by both informational validation and value-based interpretation.

Based on the above discussion, within the study we put forward the following hypothesis:

**H2.**  *Perceived credibility of online reviews has a significant positive effect on attitudes toward sustainable tourism.*

### 2.3. Social Media Platforms and Behavioral Intention Toward Sustainable Tourism

The relationship between social media platforms and behavioral intention is theoretically complex and remains debated in the literature. The TPB suggests that behavioral intention is mainly driven by attitudes. However, digital environments introduce additional pathways that can influence behavior.

From a signaling perspective, social media platforms function as environments where aggregated user feedback conveys information about service quality and social approval (Litovka-Demenina & Koval, 2025). At the same time, from a cognitive processing perspective, these platforms enable both direct (heuristic-based) and indirect (attitude-mediated) influence mechanisms.

Empirical findings reflect this duality. Some studies report strong direct effects of social media exposure on behavioral intention, driven by emotional engagement, visual appeal, and social influence (H. Wang & Yan, 2022; Prayag et al., 2013; Hudson et al., 2015). Others emphasize indirect effects mediated through constructs such as trust, perceived usefulness, or attitude (Kong et al., 2020; Hu et al., 2025). These mixed findings suggest that digital influence operates through multiple parallel pathways rather than a single linear mechanism.

In sustainable tourism contexts, this complexity is further amplified. Behavioral intention is shaped not only by exposure to information. It is also influenced by credibility, sustainability perceptions, and cognitive evaluation processes (Chen et al., 2023; Ogiemwonyi et al., 2023; Mim et al., 2022; Karsokiene et al., 2025). As a result, social media platforms may influence behavior in two ways. They can directly trigger heuristic responses and indirectly shape attitudes through credibility-based mechanisms.

Based on this integrated perspective, the study proposes that social media platforms have both direct and indirect effects on behavioral intention. This reflects the coexistence of heuristic and systematic processing pathways.

On this basis, the following hypothesis is advanced:

**H3.**  *The use of social media platforms has a significant positive effect on tourists’ behavioral intention to choose sustainable tourism options.*

### 2.4. Attitude Toward Sustainable Tourism and Behavioral Intention

The relationship between attitude and behavioral intention is a core component of the TPB and has been extensively validated in tourism research (Han et al., 2010; C. Wang et al., 2018; Q. Zhang et al., 2024; X. Wang et al., 2024; Liu et al., 2022). However, the strength and stability of this relationship depend on contextual and cognitive factors.

In sustainable tourism contexts, attitudes are more than simple evaluations. They are often linked to moral values, environmental concern, and identity-based motivations (Yadav & Pathak, 2017). This strengthens the attitude–intention relationship, as individuals are more likely to act in ways that are consistent with their internalized values.

At the same time, this relationship may be moderated by contextual factors such as social norms, perceived behavioral control, and information environment characteristics (Wut et al., 2023; Yuliawan et al., 2023; L. Wang et al., 2025). In digital environments, repeated exposure to socially validated sustainability-related content can reinforce attitudes and increase their translation into behavioral intention.

From a cognitive perspective, attitudes formed through systematic processing are more stable. They are also more predictive of behavior than those formed through heuristic processing (Petty & Cacioppo, 1986). This suggests that the strength of the attitude–intention relationship depends on the depth of cognitive evaluation.

Therefore, the following hypothesis is put forward:

**H4.**  *Tourists’ attitudes toward sustainable tourism choices have a significant positive effect on behavioral intention toward sustainable tourism choices.*

### 2.5. The Mediating Role of Environmental Awareness

Environmental awareness represents a cognitive mechanism that bridges the gap between information and behavior. Unlike attitude, which reflects evaluative judgment, environmental awareness captures knowledge-based understanding and issue-specific cognition related to environmental sustainability (Aman et al., 2021; Üzülmez et al., 2023; Chiu et al., 2014).

From a theoretical perspective, environmental awareness functions as a cognitive translation mechanism. It allows externally acquired information to be internalized and processed. This distinguishes awareness from attitude, as awareness reflects comprehension and information processing, whereas attitude represents evaluative preference. Importantly, this distinction suggests that awareness may operate independently of attitude. It can also function as an intermediate stage before evaluation.

When tourists perceive online reviews as credible, they are more likely to engage in systematic information processing rather than heuristic-based evaluation. This deeper cognitive engagement enhances their awareness of environmental issues and strengthens their ability to interpret sustainability-related information (Pourhossein et al., 2023; Filieri et al., 2015). As a result, environmental awareness helps transform credibility into behavior. It enables individuals to internalize sustainability-related cues.

However, unlike attitude, which directly drives behavioral intention, environmental awareness plays an indirect role. It shapes the cognitive basis of behavioral decisions. This suggests that awareness operates as a mediating mechanism rather than a direct predictor, linking credibility to behavior through cognitive processing pathways.

Accordingly, environmental awareness can be conceptualized as a mechanism through which perceived credibility is translated into behavioral intention. More specifically, the proposed mediation mechanism theoretically assumes that perceived credibility strengthens environmental awareness by encouraging deeper cognitive engagement with sustainability-related information, while increased environmental awareness subsequently enhances individuals’ intention to engage in sustainable tourism behavior. Thus, the mediation process reflects a sequential cognitive translation pathway linking credibility-based evaluation with sustainability-oriented behavioral outcomes.

Drawing on the above arguments, the following hypothesis is developed:

**H5.**  *Environmental awareness mediates the relationship between perceived credibility of online reviews and behavioral intention toward sustainable tourism choices.*

### 2.6. The Moderating Role of Cognitive Processing Bias

Cognitive Processing Bias (CPB) represents a systematic deviation from objective and analytical information evaluation. This concept is based on the Elaboration Likelihood Model (ELM) and the Heuristic–Systematic Model (HSM). CPB reflects individuals’ tendency to rely on heuristic rather than systematic processing when evaluating information (Petty & Cacioppo, 1986; Chaiken, 1980). Importantly, ELM and HSM distinguish between two fundamentally different processing modes:

- Systematic (central) processing: effortful, analytical, and based on argument quality
- Heuristic (peripheral) processing: simplified, cue-based, and reliant on cognitive shortcuts

Digital environments are characterized by information overload, time constraints, and algorithmic filtering. Under these conditions, individuals are more likely to engage in heuristic processing (Dwivedi et al., 2021). This increases susceptibility to cognitive bias, where judgments are influenced by superficial cues such as popularity, emotional appeal, or source attractiveness.

However, while heuristic cues are widely present across digital environments, individuals differ substantially in the extent to which they rely on such cues during information evaluation. In this regard, the present study conceptualizes Cognitive Processing Bias not as the mere existence of heuristic processing within digital environments, but as an individual-level tendency reflecting susceptibility to simplified, selective, and low-effort evaluation strategies under identical informational conditions.

Within this framework, CPB is defined as a tendency toward heuristic and selective information processing that reduces cognitive effort and limits critical evaluation. This distinguishes CPB from general cognitive limitations by focusing specifically on systematic deviations in information evaluation behavior. Theoretically, CPB acts as a boundary condition in the credibility–attitude–behavior pathway. When cognitive bias is low, individuals are more likely to process information systematically, allowing credibility to exert a stronger influence on attitude formation. In contrast, when cognitive bias is high, individuals rely more heavily on heuristic cues, weakening the translation of credibility into attitude.

This mechanism helps explain why the effects of credibility on attitudes and behavioral outcomes may vary across individuals and contexts in previous studies. In some cases, credibility does not lead to stable attitudes or behaviors. By incorporating CPB, the model explains this variability as a function of cognitive processing differences rather than inconsistencies in informational effects.

Accordingly, the credibility–attitude–behavior relationship is expected to be contingent upon the level of cognitive processing bias.

Building on the above arguments, the following hypothesis is developed:

**H6.**  *Cognitive processing bias conditionally influences the indirect effect of perceived credibility of online reviews on behavioral intention through attitude toward sustainable tourism. This indirect effect becomes weaker when cognitive processing bias is high.*

## 3. Materials and Methods

This study adopts a quantitative research design to examine relationships in the context of sustainable tourism. It focuses on social media platforms (SMP), perceived credibility of online reviews (PCOR), attitude toward sustainable tourism (AST), environmental awareness (EA), and behavioral intention (BI). The research is grounded in the Theory of Planned Behavior and e-WOM literature and aims to empirically test the proposed conceptual model.

Data were collected using a structured questionnaire based on validated measurement scales adapted from prior tourism, consumer behavior, and sustainability research. The survey was conducted in 2025 and targeted individuals with prior experience using social media platforms for travel-related information search and decision-making. It particularly focused on those engaging with online reviews related to sustainable tourism services.

To ensure content validity and contextual relevance, the questionnaire was pilot tested with a small group of respondents familiar with tourism-related digital platforms. Participants evaluated the clarity, relevance, and comprehensibility of the items, and minor modifications were made to improve wording and ensure accurate construct representation.

In this study, social media platforms are conceptualized as interactive digital environments that facilitate information exchange, user-generated content, and peer influence. Within this context, online reviews serve as key informational inputs, while their perceived credibility determines the extent to which such information is accepted and utilized in decision-making processes.

### 3.1. Measurement Instruments

The research model includes five constructs, each measured using multi-item reflective scales. All items were assessed using a five-point Likert scale ranging from 1 (strongly disagree) to 5 (strongly agree).

SMP refer to the extent to which individuals use and engage with social media platforms. This includes searching for and evaluating tourism-related information, exposure to online reviews, and interaction with travel-related content.

PCOR reflects individuals’ perceptions of the reliability, accuracy, objectivity, and trustworthiness of online reviews, particularly in evaluating tourism services and eco-friendly options.

AST represents individuals’ overall evaluation of environmentally responsible tourism practices and the extent to which such choices align with their values.

EA refers to individuals’ knowledge and understanding of environmental issues related to tourism, including awareness of environmental impacts and familiarity with sustainable tourism practices. While environmental awareness reflects cognitive understanding, it is conceptually distinct from attitude, which captures evaluative judgment.

BI represents individuals’ intention to engage in sustainable tourism behaviors, including choosing, prioritizing, and recommending eco-friendly tourism options.

CPB reflects individuals’ tendency to rely on heuristic, simplified, and selective information processing when evaluating online content. This leads to reduced cognitive effort, limited information verification, and greater reliance on superficial cues such as popularity or general impressions.

The specific measurement items used to operationalize each construct are presented in Appendix A.

### 3.2. Research Framework

Consistent with the research objectives, the conceptual framework was developed to capture the hypothesized relationships among the study variables. It includes both direct and indirect relationships. Social media platforms directly influence perceived credibility and behavioral intention. Perceived credibility, in turn, affects behavioral intention indirectly through attitude and environmental awareness.

Attitude toward sustainable tourism is treated as a core TPB construct directly influencing behavioral intention. Environmental awareness is incorporated as an additional cognitive mechanism explaining how credible information is internalized.

Cognitive processing bias is introduced as a moderating variable. It influences the strength of the indirect relationship between perceived credibility and behavioral intention through attitude.

**Figure 1:**
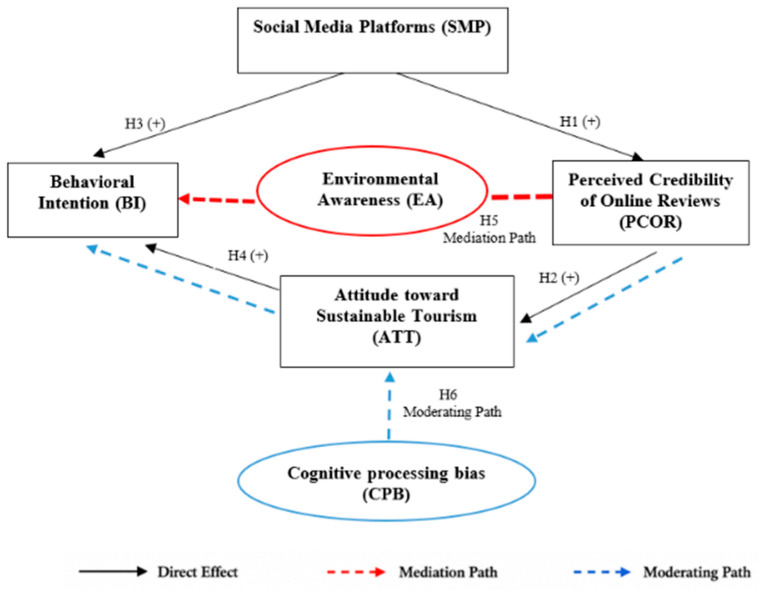
Conceptual framework of the study.

The proposed model is presented in Figure 1.

Figure 1 illustrates the hypothesized structural model of the study and the expected relationships among the key constructs. Social Media Platforms are conceptualized as the primary exogenous variable. They directly influence the perceived credibility of online reviews (H1) and also affect tourists’ behavioral intention toward sustainable tourism choices (H3). Perceived credibility is further expected to positively influence tourists’ attitudes toward sustainable tourism (H2), reflecting the role of credible information in shaping evaluative judgments.

Attitude toward sustainable tourism is positioned as a core construct derived from the Theory of Planned Behavior. It directly influences behavioral intention (H4), based on the assumption that favorable evaluations increase the likelihood of pro-environmental travel behavior.

Environmental Awareness is incorporated as a mediating variable explaining how perceived credibility of online reviews is translated into behavioral intention. Specifically, H5 proposes that higher levels of perceived credibility enhance environmental awareness, which in turn strengthens tourists’ behavioral intention toward sustainable tourism choices. The nature of this mediation (full or partial) is determined empirically based on the significance of direct and indirect effects.

The “+” signs in the figure indicate the hypothesized positive direction of all structural relationships. These relationships are tested using Structural Equation Modeling (SEM) to examine both direct and indirect effects.

Furthermore, the model includes a conditional effect (H6). Cognitive processing bias moderates the indirect relationship between perceived credibility and behavioral intention through attitude. This implies that the strength of the mediation effect depends on individuals’ cognitive processing tendencies.

### 3.3. Sampling and Data Collection

Data were collected between June and October 2025 through an online survey distributed via social media platforms and email networks in Azerbaijan. The study focused on individuals who actively use digital platforms for tourism-related information search, reflecting the increasing digitalization of tourism behavior in emerging markets.

A non-probability convenience sampling approach was employed. It targeted respondents with prior experience in searching for tourism-related information, particularly sustainable and eco-friendly tourism services. This approach is widely used in behavioral and tourism research, especially in studies aimed at testing theoretical relationships within SEM frameworks rather than achieving population representativeness.

To ensure the relevance of the sample, respondents were required to meet two screening criteria: (1) active use of social media platforms (e.g., Instagram, Facebook, TikTok), and (2) prior exposure to tourism-related content or online reviews. These criteria ensured that participants possessed sufficient experience to evaluate online information credibility and form attitudes toward sustainable tourism.

Prior to the main data collection, a pilot study involving 35 respondents was conducted to assess the clarity, reliability, and content validity of the measurement items. Based on the pilot results, minor wording revisions were implemented to enhance item clarity and ensure conceptual consistency. Reliability analysis confirmed that all constructs met acceptable internal consistency thresholds (Cronbach’s α > 0.70), and exploratory factor analysis supported the adequacy of factor loadings. The pilot data were excluded from the final analysis.

The final survey instrument consisted of multiple sections corresponding to the main constructs of the study: SMP, PCOR, AST, BI, and EA. All items were measured using a five-point Likert scale ranging from 1 (“strongly disagree”) to 5 (“strongly agree”).

After screening for completeness and response consistency, a total of 315 valid responses were retained for analysis. The final sample demonstrates sufficient variation in demographic and behavioral characteristics, supporting the robustness of the empirical analysis (see Table 1).

The sample comprises 315 respondents, with a slightly higher proportion of females (53.0%) than males (47.0%). The majority are aged between 18 and 35 years (63.5%), indicating a predominantly young and digitally active cohort. Most participants hold at least a bachelor’s degree (48.3%), followed by master’s degrees (27.9%), reflecting a relatively well-educated sample.

In terms of employment status, employed individuals represent the largest group (50.2%), followed by students (29.2%). Regarding digital engagement, a substantial proportion of respondents spend between 1–3 h (40.0%) or 3–5 h (29.8%) per day on social media. Income distribution indicates that the majority fall within the middle-income category (41.9%), which is relevant for analyzing behavioral intentions toward sustainable tourism choices.

### 3.4. Data Analysis Procedures

Data analysis was conducted using a multi-stage approach to ensure the robustness and validity of the empirical results. Statistical analyses were performed using SPSS (version 26) and AMOS (version 24).

First, descriptive statistics were computed to summarize the demographic characteristics of the respondents. Subsequently, reliability analysis was performed to assess the internal consistency of the measurement scales. Cronbach’s alpha (α) and composite reliability (CR) values were calculated for all constructs, with threshold values of 0.70 indicating acceptable reliability.

To evaluate convergent validity, standardized factor loadings and average variance extracted (AVE) were examined. Factor loadings exceeding 0.60 were considered acceptable, while values above 0.70 were regarded as ideal. AVE values above 0.50 indicated adequate convergent validity.

Discriminant validity was assessed using both the Fornell–Larcker criterion and the Heterotrait–Monotrait ratio (HTMT). According to the Fornell–Larcker criterion, the square root of AVE for each construct should exceed its correlations with other constructs. In addition, HTMT values below 0.85 confirm discriminant validity.

A confirmatory factor analysis (CFA) was conducted to validate the measurement model and ensure that the observed variables appropriately represent their respective latent constructs (SMP, PCOR, AST, BI, and EA). Model fit was evaluated using multiple fit indices, including χ^2^/df, CFI, TLI, RMSEA, and SRMR, with commonly accepted threshold values (e.g., CFI and TLI > 0.90, RMSEA < 0.08).

Following validation of the measurement model, structural equation modeling (SEM) was employed. It was used to test the hypothesized relationships and estimate both direct and indirect effects.

To test the moderating effect of cognitive processing bias (CPB) and the conditional indirect effect (H6), an interaction term was created between PCOR and CPB. This term was included in the structural model to examine whether CPB moderates the relationship between PCOR and AST.

Furthermore, a moderated mediation analysis was conducted using a bootstrapping procedure.

It assessed whether the indirect effect of PCOR on BI through AST varies across different levels of CPB. Conditional indirect effects were estimated at low, medium, and high levels of CPB using bias-corrected confidence intervals.

To examine the mediating role of EA, a bootstrapping procedure with 5000 samples was implemented in AMOS to estimate indirect effects and their statistical significance. Bias-corrected 95% confidence intervals were used, and mediation was considered significant when the confidence interval did not include zero.

### 3.5. Common Method and Non-Response Bias Assessment

Given the cross-sectional design and reliance on self-reported data, several procedural and statistical remedies were applied to assess potential common method variance (CMV). First, procedural remedies were implemented during the questionnaire design stage. These included ensuring respondent anonymity and minimizing item ambiguity to reduce evaluation apprehension and method bias.

Second, a common latent factor (CLF) was incorporated into the confirmatory factor analysis. All measurement items were allowed to load on both their theoretical constructs and the common latent factor. The results showed that including the CLF did not produce substantial changes in standardized factor loadings or structural path estimates. All differences remained below the recommended threshold of 0.20 (maximum observed difference < 0.10). This suggests that CMV is unlikely to significantly bias the results.

The results of the CLF test are presented in Table 2.

The differences in standardized loadings remain below the threshold of 0.20, further indicating that common method bias is not a concern.

Third, Harman’s single-factor test was conducted using exploratory factor analysis. The findings showed that the largest single factor accounted for less than 50% of the total variance. This provides additional evidence that CMV is not a serious concern in this study.

To further ensure robustness, non-response bias was assessed by comparing early and late respondents using independent-sample *t*-tests across the main constructs (SMP, PCOR, AST, BI, and EA). The results revealed no statistically significant differences (*p* > 0.05), indicating that non-response bias is unlikely to affect the validity of the results.

Multicollinearity was assessed using variance inflation factors (VIF), with results presented in Table 3.

All VIF values are below the conservative threshold of 3.3, indicating that multicollinearity is not a concern in the model.

### 3.6. Reliability and Validity Analyses

Empirical analyses were conducted using IBM SPSS Statistics (version 26) and IBM AMOS (version 24). Prior to the main analysis, all measurement items were examined for factor loadings. Items with loadings below the recommended threshold of 0.60 were considered for removal; however, all retained items satisfied this criterion.

The measurement model was evaluated in terms of reliability and convergent validity. As presented in Table 4, all constructs demonstrate satisfactory internal consistency.

These include SMP, PCOR, AST, BI, EA, and CPB. Cronbach’s alpha (α) and composite reliability (CR) values exceed the recommended threshold of 0.70, indicating acceptable to strong reliability.

To assess convergent validity, standardized factor loadings and average variance extracted (AVE) were examined. All factor loadings exceed 0.60, and AVE values meet or exceed the recommended threshold of 0.50. This indicates that the constructs adequately capture the variance of their respective indicators.

Discriminant validity was assessed using both the Fornell–Larcker criterion and the heterotrait–monotrait (HTMT) ratio. As presented in Table 5, the square roots of AVE values (diagonal elements) are greater than the corresponding inter-construct correlations. This indicates satisfactory discriminant validity.

Additionally, HTMT values reported in Table 6 are below the threshold of 0.85, providing further evidence that the constructs are empirically distinct.

The results of the confirmatory factor analysis (CFA) indicate that the measurement model exhibits an excellent overall fit. The key fit indices fall well within the recommended thresholds (χ^2^/df = 1.335, RMSEA = 0.029, CFI = 0.986, TLI = 0.920, GFI = 0.914, NFI = 0.973, SRMR = 0.034), suggesting that the model adequately represents the underlying latent constructs (see Table 7).

Additional diagnostics using a common latent factor approach confirmed that common method variance is not a concern. Differences in standardized loadings remained below 0.20.

The inclusion of Cognitive Processing Bias enhances the explanatory depth of the model.

It captures cognitive distortions in information processing and enables the examination of conditional effects within a moderated mediation framework.

## 4. Results

Structural equation modeling (SEM) was employed to test the hypothesized relationships. Both mediating and moderating mechanisms were examined to provide a comprehensive understanding of the underlying decision-making processes.

The mediating role of EA was assessed using a bootstrapping procedure with 5000 resamples. Furthermore, to test the moderated mediation hypothesis (H6), an interaction term (PCOR × CPB) was incorporated into the model. This was used to examine whether Cognitive Processing Bias conditions the indirect effect of perceived credibility on behavioral intention through attitude.

**Figure 2:**
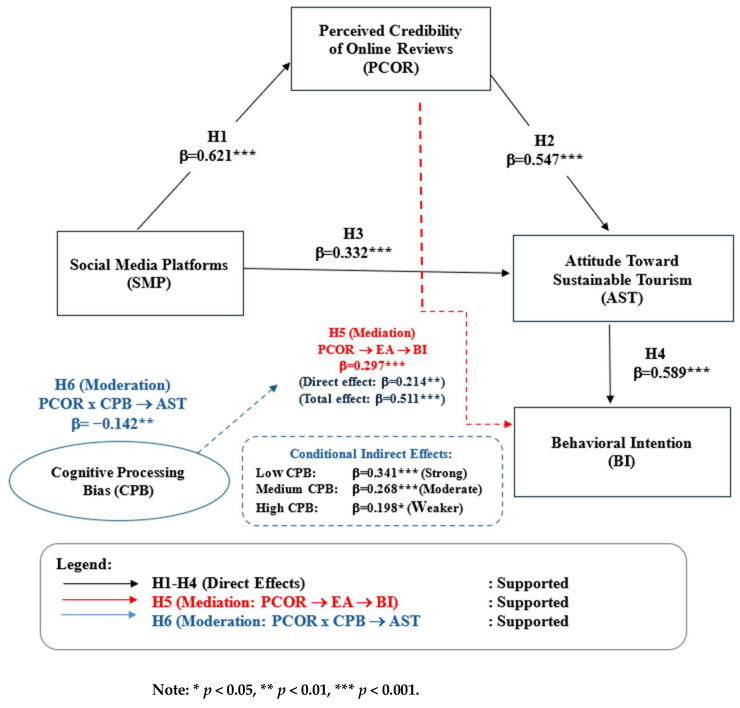
Structural equation modeling results (standardized path coefficients).

The results of the structural model are presented in Table 6, Table 7 and Table 8. These include standardized path coefficients (β), standard errors, critical ratios, and significance levels. The overall model is illustrated in Figure 2.

Table 8 presents the estimated direct structural relationships (H1–H4). All hypothesized paths are positive and statistically significant (*p* < 0.001).

In addition to path coefficients, the explanatory power of the model was assessed using R^2^ values. The model explains a substantial proportion of variance in the key endogenous constructs. Specifically, social media platforms explain 40% of the variance in PCOR (R^2^ = 0.40). Perceived credibility explains 35% of the variance in AST (R^2^ = 0.35). Furthermore, the combined effects of social media platforms, attitude, and environmental awareness explain 60% of the variance in BI (R^2^ = 0.60), indicating satisfactory explanatory power of the structural model.

These results indicate that evaluative mechanisms play a dominant role in shaping behavioral intention. Social media influence operates both directly and indirectly through credibility and attitudinal pathways.

The effect of perceived credibility on attitude (β = 0.547) can be characterized as moderate to strong, highlighting the important role of credible information in attitude formation. In contrast, the direct effect of social media platforms on behavioral intention (β = 0.332) is moderate, indicating that digital influence operates both directly and indirectly through mediating mechanisms.

As shown in Table 9, environmental awareness partially mediates the relationship between perceived credibility and behavioral intention. Both the direct (β = 0.214, *p* < 0.01) and indirect effects (β = 0.297, *p* < 0.001) are statistically significant, confirming partial mediation.

In terms of effect size, the indirect effect (β = 0.297) can be considered moderate, while the direct effect (β = 0.214) is relatively weaker.

Table 10 reports the results of the moderated mediation analysis.

The interaction effect between perceived credibility and cognitive processing bias is negative and statistically significant (β = −0.142, *p* < 0.01). This indicates that cognitive processing bias weakens the relationship between perceived credibility and attitude.

The conditional indirect effects decrease across levels of cognitive processing bias—from low (β = 0.341) to medium (β = 0.268) and high (β = 0.198)—demonstrating that higher levels of bias reduce the strength of the mediating mechanism. Specifically, the indirect effect is moderate to strong at low levels of bias, moderate at medium levels, and weak to moderate under high levels of bias. These findings support H6.

## 5. Discussion

The findings of this study provide important insights into how digital information environments shape sustainable tourism decision-making. Rather than operating through a single linear pathway, the results reveal a multi-layered mechanism. In this mechanism, social media exposure, perceived credibility, cognitive evaluation, and behavioral intention interact simultaneously.

### 5.1. The Influence of Social Media Platforms on the Perceived Credibility of Online Reviews

The findings indicate a strong positive relationship between social media platform usage and the perceived credibility of online reviews (H1), which can be explained by the structural and interactive nature of digital platforms. Unlike traditional information channels, social media environments facilitate social validation mechanisms—such as likes, comments, shares, and peer feedback—which function as heuristic cues in credibility assessment.

From a theoretical perspective, the results suggest that individuals rely not only on content quality but also on social endorsement signals when evaluating information credibility. This is particularly evident in uncertain contexts such as sustainable tourism.

In such cases, environmental claims are difficult to verify directly, so users rely more on collective intelligence and peer-generated signals.

The findings further demonstrate that algorithm-driven content exposure reinforces repeated interaction with similar types of information. This increases familiarity and perceived trustworthiness. This indicates that credibility is shaped not only by objective information quality but also by platform-induced cognitive heuristics and exposure effects. In this sense, social media platforms operate as credibility amplification systems that influence how users interpret and internalize tourism-related content.

This result is consistent with earlier studies emphasizing the role of platform interactivity and user-generated content in shaping credibility perceptions (Ayeh et al., 2013; Filieri et al., 2018; Guzzo et al., 2022). At the same time, the present findings provide additional insights that extend this literature.

First, while prior research has primarily examined online review credibility in general tourism or accommodation contexts, the findings demonstrate that credibility remains equally critical in sustainable tourism decisions, where claims are inherently more difficult to verify. Second, the results suggest that credibility is not solely embedded in the message itself. It is co-produced within the broader social media environment, including platform architecture, peer interaction, and collective endorsement.

### 5.2. Perceived Credibility and Attitudes Toward Sustainable Tourism

The findings indicate that the positive effect of perceived credibility on attitudes toward sustainable tourism (H2) reflects the role of trust-based cognitive processing in attitude formation. When individuals perceive online reviews as credible, they are more likely to engage in deeper cognitive evaluation, leading to the internalization of sustainability-related information. This is consistent with prior findings suggesting that the perceived credibility of online reviews enhances information acceptance and shapes evaluative responses through cognitive processing mechanisms (Chong et al., 2018; Kaur & Kaur, 2023). However, previous studies emphasize information adoption as a primarily rational process.

In contrast, the present findings suggest that in sustainability contexts credibility operates more as a legitimizing mechanism rather than purely an informational one.

This relationship can be further explained through uncertainty reduction theory. Sustainable tourism often involves abstract and non-observable attributes (e.g., environmental responsibility, eco-certification). In such contexts, the results suggest that credible online reviews function as surrogate verification mechanisms, reducing ambiguity and increasing confidence in decision-making. This interpretation is aligned with trust-based evaluation frameworks, which emphasize that credibility reduces perceived risk and enhances confidence in complex decision environments (Roos, 2024; Su et al., 2022; Zelenka et al., 2021). Unlike these studies, which focus on trust formation in general tourism contexts, the present results highlight a different pattern. Credibility plays a more critical role in evaluating sustainability claims, where verification is inherently more difficult.

Importantly, the findings show that credibility does not merely inform attitudes—it legitimizes sustainability claims. Without perceived credibility, sustainability-related messages may be dismissed as greenwashing. Thus, credibility operates as a filtering mechanism through which information is accepted and validated. It also translates information into positive evaluative judgments, explaining its strong influence on attitudes in sustainability-oriented tourism decisions.

This legitimizing role appears particularly critical in sustainability communication, where environmental narratives must overcome skepticism and perceived ambiguity to influence consumer evaluations. This interpretation is consistent with studies emphasizing the importance of credibility in sustainability-related communication contexts (Rahman & Mia, 2025; Rao et al., 2025).

While this result supports prior research linking credible online information with favorable tourism evaluations, it also refines that literature. Earlier studies have primarily treated credibility as a precursor to trust, usefulness, or adoption, with limited attention to its role in sustainability-specific attitude formation (Ayeh et al., 2013; Filieri, 2015; Sadik-Zada et al., 2024). Additional evidence also suggests that the structure and presentation of online reviews can influence how credibility translates into consumer evaluations and attitudes (Bae & Lee, 2011; Nguyen, 2025).

In contrast, the present findings indicate that credibility performs a stronger legitimizing function in sustainable tourism contexts. Tourists rely on credible reviews not only to reduce uncertainty but also to assess the validity of sustainability claims. This suggests that credibility should be understood not simply as an informational filter, but as an evaluative mechanism through which sustainability-related messages become attitude-relevant.

### 5.3. Attitudes and Behavioral Intention

The findings confirm a strong relationship between attitudes toward sustainable tourism and behavioral intention (H4), consistent with the TPB. However, the magnitude of this relationship in the present study suggests the presence of additional underlying mechanisms. Specifically, sustainability-related attitudes appear to be closely linked to moral values and personal identity, making them more likely to translate into intention compared to purely utilitarian attitudes. This interpretation is supported by prior research indicating that environmentally oriented attitudes are strongly associated with value-driven behavioral intentions in tourism contexts (Juvan & Dolnicar, 2016; X. Zhang & Dong, 2020).

In this context, choosing sustainable tourism options reflects not only a rational decision but also a form of value expression and self-consistency behavior. The results suggest that individuals with favorable sustainability attitudes tend to align their behavioral intentions with their internalized environmental values in order to maintain cognitive consistency. This value-driven alignment is consistent with models of pro-environmental behavior, where internal motivations and cognitive evaluations jointly shape behavioral outcomes (J. J. Li et al., 2023; Jiang et al., 2022; Zheng et al., 2025).

Furthermore, the findings indicate that social desirability and normative expectations reinforce this relationship. Pro-environmental behavior is increasingly perceived as socially responsible. This suggests that attitudes toward sustainable tourism function not only as cognitive evaluations but also as normatively supported drivers of intention, thereby strengthening their predictive power. This interpretation aligns with research highlighting the role of social and contextual influences in shaping environmentally responsible tourism behavior (Zhou et al., 2023; Težak Damijanić et al., 2023).

The results are consistent with prior TPB-based sustainable tourism studies reporting a positive attitude–intention relationship (Han et al., 2010; C. Wang et al., 2018; Wut et al., 2023). At the same time, the present findings extend this literature by demonstrating that this relationship remains particularly strong within digitally mediated environments shaped by online reviews and social media exposure. In this context, digital environments appear to reinforce the translation of attitudes into behavioral intention by continuously shaping sustainability perceptions and decision contexts (Nguyen, 2025; Rahman & Mia, 2025).

Importantly, the findings suggest that attitudes are not formed in isolation but emerge within an informational environment enriched by peer-generated sustainability narratives. This may explain the robustness of the attitude–intention link observed in the model, where positive evaluations are reinforced by both internal values and socially transmitted digital cues.

### 5.4. Direct Effects of Social Media Platforms and Mediation Mechanism

The direct effect of social media platforms on behavioral intention (H3) suggests an important insight. Decision-making in digital environments is not exclusively mediated by cognitive evaluation processes. While prior literature may overemphasize direct platform effects, the present findings indicate that behavioral intention is more strongly shaped through credibility and attitudinal pathways. This is consistent with research suggesting that social media environments influence behavioral outcomes through both direct exposure effects and indirect cognitive mechanisms (D. Leung et al., 2013; Mariani et al., 2016).

Although the Theory of Planned Behavior emphasizes attitudinal mechanisms, the results support a dual-process perspective in which behavioral intention is influenced by both cognitive (attitude-based) and heuristic (emotion- or exposure-driven) processes. Such dual mechanisms are also highlighted in studies of pro-environmental behavior, which emphasize the coexistence of rational evaluation and affective or heuristic influences in shaping behavioral responses (Cao et al., 2022; Khan et al., 2021) Unlike linear decision-making models that assume intention is fully transmitted through attitudes, the findings demonstrate that digital environments enable parallel influence mechanisms.

Social media platforms provide instant, visually rich, and emotionally engaging content that may stimulate rapid and heuristic-based evaluations. As a result, individuals may develop behavioral tendencies without fully engaging in systematic information processing, which helps explain the persistence of a significant direct effect even in the presence of mediators. This aligns with evidence suggesting that digital tourism environments can stimulate immediate and emotion-driven responses through exposure to persuasive and experience-based content (Nguyen, 2025; Rao et al., 2025).

Accordingly, the results diverge from strictly linear interpretations of tourism decision-making. Rather than operating through a single pathway, social media influences intention both directly and indirectly by creating immediacy, visibility, and social momentum that shape tourism-related evaluations and decisions. This layered mechanism indicates that sustainable tourism intention is formed through the combined effects of platform exposure, credibility assessment, and internal psychological processing.

Importantly, the direct SMP → BI relationship should not be interpreted as a purely heuristic or impulsive effect. Rather, it may reflect additional parallel mechanisms not fully captured within the current model, including repeated digital exposure, social reinforcement, platform engagement intensity, and normative influence embedded within social media environments. Accordingly, the direct effect represents a broader informational and contextual influence of digital platforms beyond the credibility- and attitude-based pathways explicitly modeled in the study.

Regarding mediation, environmental awareness was found to partially mediate the relationship between perceived credibility and behavioral intention (H5). The presence of both a significant indirect effect and a remaining direct effect (β = 0.214, *p* < 0.01) indicates partial mediation, suggesting that credibility influences intention through multiple parallel pathways. This finding is consistent with studies indicating that environmental awareness enhances behavioral intention but does not fully account for the complexity of pro-environmental decision-making processes (Jiang et al., 2022; Zhou et al., 2023).

One pathway operates through environmental awareness, where credible information enhances individuals’ understanding of environmental issues and strengthens intention through cognitive processing. At the same time, credibility exerts an independent influence by increasing trust, reducing perceived risk, and enhancing perceived value. This multi-path influence is also supported by research emphasizing that credibility and trust-based evaluations independently contribute to tourism-related behavioral outcomes (Zelenka et al., 2021; Su et al., 2022). This indicates that environmental awareness captures only one dimension of the underlying mechanism. Additional pathways—such as trust in sustainability claims, perceived authenticity, or value congruence—may also contribute to behavioral intention. Therefore, the mediation results reflect a multi-layered decision-making process in which both cognitive (awareness-based) and evaluative (trust-based) mechanisms operate simultaneously.

From a theoretical perspective, this partial mediation is particularly important. Contrary to models that treat environmental awareness as a sufficient explanatory bridge between information and behavior, the findings demonstrate that awareness is consequential but not exhaustive. This is in line with broader literature suggesting that pro-environmental behavior is shaped through multiple interacting cognitive and contextual factors rather than a single mediating mechanism (J. Zhang et al., 2024; Carvajal-Trujillo et al., 2024) Credible online reviews influence behavioral intention not only through increased environmental cognition but also through alternative channels related to trust and perceived value.

Thus, environmental awareness should be conceptualized as one component within a broader multi-path decision structure, rather than as a standalone explanatory mechanism.

### 5.5. The Role of Environmental Awareness in Sustainable Tourism Decisions

The findings indicate that environmental awareness plays a critical role in bridging the gap between information and behavior. It enhances individuals’ ability to interpret and apply sustainability-related knowledge. Unlike attitude, which reflects evaluative judgment, environmental awareness represents cognitive understanding and issue-specific knowledge, enabling more informed decision-making. This is consistent with prior research emphasizing that environmental awareness functions as a key cognitive driver of pro-environmental behavior by enhancing individuals’ understanding of sustainability-related issues (Jiang et al., 2022; J. J. Li et al., 2023).

The results further suggest that awareness does not automatically translate into positive attitudes or behavior; rather, it enhances the cognitive processing capacity through which individuals evaluate information. This helps explain why environmental awareness strengthens—but does not fully determine—behavioral intention in the present model. Such an interpretation is supported by literature indicating that environmental knowledge and awareness require additional psychological and contextual conditions to produce behavioral change (X. Zhang & Dong, 2020; Dolnicar et al., 2008).

In the context of social media, the findings demonstrate that higher environmental awareness enables users to better distinguish between credible and non-credible content, thereby improving decision quality. This is in line with studies suggesting that informed users are more capable of critically evaluating online information and identifying authentic sustainability-related content (Nguyen, 2025; Zelenka et al., 2021).

Importantly, the results show that environmental awareness functions as a cognitive amplifier, strengthening the effect of credible information on behavioral intention. The observed partial mediation effect confirms that awareness facilitates the translation of information into behavior while allowing additional mechanisms to operate simultaneously. This interpretation is consistent with studies indicating that environmental awareness enhances—but does not fully determine—behavioral intention, reflecting the presence of multiple parallel decision pathways (Zhou et al., 2023; Težak Damijanić et al., 2023).

At the same time, the findings highlight that awareness alone is insufficient to fully explain behavioral outcomes and should be considered alongside attitudinal and affective factors. This helps clarify a recurring ambiguity in prior literature, where environmental awareness is often conflated with related constructs such as attitude, concern, or knowledge. Existing research also emphasizes the importance of distinguishing cognitive awareness from evaluative and affective constructs in explaining sustainable tourism behavior (Carvajal-Trujillo et al., 2024; Lemy et al., 2026).

Overall, the findings support conceptualizing environmental awareness as a distinct cognitive mechanism rather than as an extension of environmental attitude. From a theoretical perspective, this study demonstrates that individuals must first cognitively recognize and understand the environmental relevance of tourism choices before these considerations become behaviorally actionable. This interpretation is further supported by literature suggesting that pro-environmental behavior emerges through the interaction of knowledge, perception, and contextual influences rather than through a single determinant (J. Zhang et al., 2024; Yaja et al., 2026).

Thus, environmental awareness should be viewed not merely as an accompanying variable, but as a key component in the translation process through which credible digital information is transformed into sustainable behavioral intention.

### 5.6. Moderated Mediation: The Role of Cognitive Processing Bias

Environmental awareness explains the cognitive pathway through which credible information influences behavioral intention. However, the moderated mediation results indicate that this mechanism is not uniform across individuals and depends on the level of cognitive processing bias.

The findings suggest that cognitive processing bias significantly weakens the relationship between perceived credibility and attitudes toward sustainable tourism. Individuals with higher levels of cognitive bias are less likely to engage in systematic evaluation of information. Instead, they rely on heuristic cues such as popularity, emotional appeal, or superficial content characteristics. As a result, even when online reviews are perceived as credible, their influence on attitude formation becomes less effective.

This finding supports a dual-process interpretation of decision-making, where both systematic (cognitive) and heuristic (bias-driven) processing mechanisms operate simultaneously. However, unlike prior studies that primarily emphasize the coexistence of these processes, the present results demonstrate that cognitive bias can actively disrupt the credibility–attitude linkage, reducing the effectiveness of credible information in shaping evaluative judgments.

In digital environments characterized by high information volume and algorithm-driven exposure, individuals are frequently exposed to simplified and repetitive content, which may reinforce heuristic processing tendencies. This suggests that the effectiveness of credible sustainability information is contingent not only on its quality but also on the cognitive processing style of the individual.

Importantly, the results indicate that cognitive processing bias does not eliminate the influence of credibility but attenuates its impact, leading to weaker attitudinal responses. This helps explain why, despite strong credibility effects observed in the model, variability exists in how individuals translate credible information into attitudes and behavioral intention.

From a theoretical perspective, these findings extend existing models of sustainable tourism behavior by introducing cognitive processing bias as a boundary condition. While previous research has largely treated credibility and awareness as stable predictors of behavior, the present results demonstrate that their effects are conditional and dependent on individual cognitive tendencies.

This moderated mediation structure highlights that sustainable tourism decision-making is not only a function of information and awareness but also of how that information is cognitively processed. Thus, the translation of credible digital information into behavioral intention should be understood as a contingent process, shaped by the interaction between information quality, cognitive evaluation, and individual-level processing bias.

## 6. Conclusions

This study examined how digital information environments shape sustainable tourism decision-making. It integrated social media usage, perceived credibility of online reviews, attitude formation, environmental awareness, behavioral intention, and cognitive processing bias into a unified structural framework. Using structural equation modeling based on 315 responses, the findings provide strong empirical evidence. They show that decision-making in digital tourism contexts operates through interconnected cognitive and evaluative mechanisms rather than a simple linear process.

The results highlight that social media platform significantly enhance the perceived credibility of online reviews, confirming their role as key informational environments in which credibility is socially constructed. Perceived credibility, in turn, exerts a substantial influence on attitude formation, indicating that credibility functions as a critical cognitive filter through which sustainability-related information is evaluated and legitimized.

Importantly, the findings demonstrate that attitude is the strongest predictor of behavioral intention, emphasizing the dominant role of evaluative mechanisms in sustainable tourism decisions. While social media platforms also exert a direct effect, this effect is comparatively moderate, suggesting that digital influence operates through both direct exposure and indirect credibility-based pathways.

The mediation analysis further reveals that environmental awareness serves as a meaningful but partial cognitive mechanism linking credibility to behavioral intention. This indicates that credibility influences behavior through multiple parallel mechanisms beyond awareness.

A key contribution of this study lies in demonstrating that these relationships are conditional rather than uniform. Cognitive processing bias significantly weakens the impact of perceived credibility on attitude and reduces the strength of the indirect effect on behavioral intention. This finding highlight that the effectiveness of credible digital information depends on individuals’ cognitive processing style. It therefore introduces an important boundary condition into sustainable tourism decision-making models.

Overall, the study advances existing literature by demonstrating that sustainable tourism decision-making cannot be fully explained by traditional linear models such as TPB alone. Instead, it provides empirical evidence that credibility-driven cognitive mechanisms and individual-level processing biases jointly determine how digital information is translated into behavior. By explicitly incorporating cognitive processing bias as a boundary condition, the study introduces a more dynamic and conditional framework for understanding sustainable tourism behavior in digital environments.

Taken together, the findings suggest that sustainable tourism decision-making in digital environments is primarily driven by credibility-based cognitive evaluation rather than mere information exposure. This highlights that the effectiveness of digital sustainability communication depends not only on the availability of information but on how that information is processed and internalized by individuals.

From a practical perspective, the findings suggest that simply increasing the volume of online sustainability-related content is insufficient to influence tourist behavior. Instead, tourism stakeholders should prioritize credibility-enhancing strategies, such as verified user-generated content, transparent sustainability claims, and platform-level credibility signals. Social media platforms should design algorithms that promote high-quality and sustainability-oriented content rather than engagement-driven visibility alone. In practical terms, tourism platform designers may implement credibility verification mechanisms such as verified reviewer badges, AI-assisted fake review detection systems, and transparency indicators for sustainability-related claims. Destination managers may also develop targeted digital campaigns emphasizing locally verified environmental practices and evidence-based sustainability communication in order to strengthen tourists’ trust and cognitive engagement. Furthermore, policymakers and destination managers should implement targeted environmental awareness programs.

These programs should enhance individuals’ capacity for systematic information processing and improve the effectiveness of sustainability communication strategies.

In essence, sustainable tourism behavior is shaped less by the quantity of digital information and more by its perceived credibility and the cognitive processes through which it is evaluated. This underscores the need to shift from information-driven to cognition-driven approaches in promoting sustainable tourism choices.

### Limitations and Future Research Directions

While the current model emphasizes the roles of perceived credibility of online reviews, environmental awareness, and cognitive processing bias, it does not explicitly incorporate several other potentially influential factors, such as perceived risk, trust in digital platforms, social influence, cultural context, and additional TPB dimensions including subjective norms and perceived behavioral control. These variables may play an important role in shaping sustainable tourism decision-making and could further strengthen the explanatory power of the proposed framework.

In addition, the findings should be interpreted with caution, as the empirical analysis is based on a relatively limited sample collected from a single-country context (Azerbaijan), which may restrict the generalizability of the results across different tourism populations and cultural settings. Future studies are encouraged to employ larger and cross-country samples, as well as examine different tourism contexts, such as urban versus rural destinations or luxury versus eco-tourism markets, in order to test the robustness and broader applicability of the proposed framework.

Furthermore, future research may benefit from applying more robust research designs, including longitudinal data, instrumental variable approaches, and advanced econometric techniques, to strengthen causal interpretation and provide a deeper understanding of cognitively mediated sustainable tourism behavior in digital environments.

## Figures and Tables

**Table 1:** Demographic Profile of Respondents.

Variable	Category	Sample Size	%	Variable	Category	Sample Size	%
Gender	Male	148	47.0	Age	18–25	102	32.4
	Female	167	53.0		26–35	98	31.1
					36–45	60	19.0
					46–55	35	11.1
					56 and above	20	6.3
Education Level	High School	48	15.2	Profession	Student	92	29.2
	Bachelor’s Degree	152	48.3		Employed	158	50.2
	Master’s Degree	88	27.9		Self-employed	32	10.2
	Doctoral Degree	27	8.6		Unemployed	21	6.7
					Other	12	3.8
Time Spent on Social Media	Less than 1 h	38	12.1	Income Level	Up to 1000 AZN	96	30.5
	1–3 h	126	40.0		Between 1000–2000 AZN	132	41.9
	3–5 h	94	29.8		More than 2000 AZN	87	27.6
	More than 5 h	57	18.1				

**Table 2:** Common Method Bias (CLF Comparison).

Construct	Without CLF (Loading)	With CLF (Loading)	Difference
SMP	0.78	0.75	0.03
PCOR	0.82	0.77	0.05
AST	0.84	0.81	0.03
EA	0.81	0.76	0.05
CPB	0.80	0.76	0.04
BI	0.86	0.83	0.03

**Table 3:** Multicollinearity Diagnostics (VIF Results).

Construct	SMP	PCOR	ATT	EA	CPB	BI
VIF	2.14	2.36	2.58	2.41	2.19	—

**Table 4:** Reliability and Validity Results.

Construct	Item	Factor Loading	Cronbach’s Alpha	Composite Reliability (CR)	AVE
Social Media Platforms	SMP1	0.794	0.842	0.887	0.608
	SMP2	0.735			
	SMP3	0.781			
	SMP4	0.806			
Perceived Credibility of Online Reviews	PCOR1	0.774	0.861	0.898	0.687
	PCOR2	0.812			
	PCOR3	0.835			
	PCOR4	0.821			
Attitude toward Sustainable Tourism	AST1	0.793	0.874	0.905	0.705
	AST2	0.842			
	AST3	0.865			
	AST4	0.821			
Behavioral Intention	BI1	0.802	0.889	0.918	0.736
	BI2	0.856			
	BI3	0.874			
	BI4	0.861			
Environmental Awareness	EA1	0.768	0.853	0.892	0.674
	EA2	0.812			
	EA3	0.835			
	EA4	0.801			
Cognitive Processing Bias	CPB1	0.780	0.74	0.811	0.695
	CPB2	0.818			
	CPB3	0.804			
	CPB4	0.719			

**Table 5:** Discriminant validity test of the model.

Construct	SMP	PCOR	AST	BI	EA	CPB
SMP	0.780					
PCOR	0.612	0.829				
AST	0.575	0.684	0.840			
BI	0.548	0.621	0.702	0.858		
EA	0.520	0.598	0.655	0.681	0.821	
CPB	0.495	0.552	0.683	0.708	0.690	0.833

**Table 6:** HTMT ratios for discriminant validity.

Construct	SMP	PCOR	AST	BI	EA	CPB
SMP	—	0.72	0.68	0.64	0.61	0.65
PCOR		—	0.76	0.71	0.69	0.70
AST			—	0.79	0.74	0.73
BI				—	0.77	0.71
EA					—	0.75
CPB						—

**Table 7:** Model Fit Indices.

Fit Index	Recommended Value	Model Value
χ^2^/df	<3.00	1.335
RMSEA	<0.08	0.029
CFI	>0.90	0.986
TLI	>0.90	0.920
GFI	>0.90	0.914
NFI	>0.90	0.973
SRMR	<0.08	0.034

**Table 8:** Results of the direct structural relationships.

Path Relationship	Path Coefficient (β)	S.E.	C.R.	*p*-Value	Hypothesis Result
SMP → PCOR	0.621	0.074	8.392	<0.001	H1 Supported
PCOR → AST	0.547	0.086	6.753	<0.001	H2 Supported
SMP → BI	0.332	0.091	3.648	<0.001	H3 Supported
AST → BI	0.589	0.079	7.456	<0.001	H4 Supported

**Table 9:** Mediation Analysis Results.

Effect Type	Path	Standardized Effect (β)	S.E.	95% Bootstrap CI	*p*-Value
Direct effect (c′)	PCOR → BI	0.214	0.087	[0.062, 0.381]	0.003
Indirect effect (a × b)	PCOR → EA → BI	0.297	0.065	[0.174, 0.438]	<0.001
Total effect (c)	PCOR → BI	0.511	0.092	[0.329, 0.693]	<0.001

**Table 10:** Moderation and Conditional Indirect Effects.

Effect	Β	S.E.	C.R.	*p*-Value	Interpretation
PCOR × CPB → AST	−0.142	0.052	−2.73	0.006	Significant (negative moderation)
Conditional indirect effect (low CPB)	0.341	0.071	—	<0.001	Strong mediation
Conditional indirect effect (medium CPB)	0.268	0.058	—	<0.001	Moderate mediation
Conditional indirect effect (high CPB)	0.198	0.063	—	0.012	Weaker mediation

## Data Availability

The original contributions presented in this study are included in the article. Further inquiries can be directed to the corresponding author.

## Appendix A

**Table A1:** Measurement items.

**Social Media Platforms (SMP)**	
	I rely on social media platforms to explore sustainable tourism options.
	I actively search for user-generated reviews on social media platforms before making tourism decisions.
	I actively engage with travel-related content on social media (e.g., likes, comments, shares).
	Social media platforms influence my perceptions about tourism services.
**Perceived Credibility of Online Reviews**	
	I believe that most online reviews reflect real customer experiences.
	I find online reviews on social media to be reliable sources of information.
	I believe that online reviews provide accurate information about eco-friendly tourism options.
	I perceive online reviews about tourism services as unbiased.
**Attitude toward Sustainable Tourism**	
	Choosing environmentally friendly tourism options is important to me.
	I believe sustainable tourism contributes to environmental protection.
	Supporting eco-friendly tourism services aligns with my personal values.
	I prefer tourism services that follow sustainable practices.
**Behavioral Intention**	
	I intend to pay more for environmentally friendly tourism services.
	I intend to choose sustainable tourism options whenever possible.
	I intend to prioritize sustainability in my tourism decisions.
	I intend to recommend sustainable tourism options to others.
**Environmental Awareness**	
	I am aware of the environmental impacts of tourism activities.
	I understand how tourism can contribute to environmental degradation (e.g., pollution, overuse of resources).
	I am informed about sustainable and eco-friendly tourism practices.
	I can distinguish between sustainable and non-sustainable tourism services.
**Cognitive processing bias**	
	I often make decisions based on general feelings rather than detailed information.
	I do not spend much time evaluating the accuracy of online reviews.
	I tend to trust reviews that are popular or highly rated, even without carefully evaluating their content.
	I rarely compare multiple sources before forming an opinion about tourism services.
